# Anti-carbamylated protein antibodies in the pre-symptomatic phase of rheumatoid arthritis, their relationship with multiple anti-citrulline peptide antibodies and association with radiological damage

**DOI:** 10.1186/s13075-015-0536-2

**Published:** 2015-02-07

**Authors:** Mikael Brink, Marije K Verheul, Johan Rönnelid, Ewa Berglin, Rikard Holmdahl, Rene EM Toes, Lars Klareskog, Leendert A Trouw, Solbritt Rantapää-Dahlqvist

**Affiliations:** Public Health and Clinical Medicine/Rheumatology, Umeå University, Umeå, SE-90185 Sweden; Department of Rheumatology, Leiden University Medical Center, Leiden, Netherlands; Department of Immunology, Genetics and Pathology, Uppsala University, Uppsala, Sweden; Department of Medical Biochemistry and Biophysics, Medical Inflammation Research, Karolinska Institute, Stockholm, Sweden; Department of Medicine, Rheumatology Unit, Karolinska Institute, Stockholm, Sweden

## Abstract

**Introduction:**

The presence of a new autoantibody system, anti-carbamylated protein (anti-CarP) antibodies, has been identified in rheumatoid arthritis (RA). The presence of anti-CarP antibodies was evaluated in samples taken from individuals who subsequently developed RA before and after onset of symptoms and related to previously analysed antibodies against citrullinated peptides (ACPA specificities) and anti-CCP2.

**Methods:**

A total of 252 individuals, with 423 samples from before onset of symptoms of RA, and 197 population controls were identified as donors to the Medical Biobank of Northern Sweden; 192 of them were also sampled at the time of diagnosis. All samples were analysed for anti-CarP IgG and anti-CCP2 antibodies using ELISAs. Ten different antibody reactivities against citrullinated antigens (ACPA specificities) were analysed using a custom-made microarray based on the ImmunoCAP ISAC system (Phadia).

**Results:**

The concentration of anti-CarP antibodies was significantly increased in the pre-symptomatic individuals compared with controls (*P* <0.001) and also increased significantly after disease onset (*P* <0.001). The sensitivity for anti-CarP antibodies in the pre-symptomatic individuals was 13.9% (95% CI: 11 to 17.6) and 42.2% (95% CI: 35.4 to 49.3) following development of RA. Anti-CarP antibody positivity was found in 5.1% to 13.3% of individuals negative for anti-CCP2 or ACPA specificities. Presence of anti-CarP antibodies was significantly related to radiological destruction at baseline, at 24 months and also to radiological change (*P* <0.05, all).

**Conclusions:**

The results indicate that anti-CarP antibodies are associated with disease development, even after adjusting for the presence of different ACPA fine specificities, and in anti-CCP2 negative individuals and contribute to the identification of a subset of patients with worse radiological progression of the disease independent of ACPA.

**Electronic supplementary material:**

The online version of this article (doi:10.1186/s13075-015-0536-2) contains supplementary material, which is available to authorized users.

## Introduction

Rheumatoid arthritis (RA) is a chronic autoimmune disease characterized by inflammation within the joints that eventually leads to the destruction of cartilage and bone. However, the aetiopathogenesis of this disease is not yet fully understood. A number of autoantibodies have been associated with the disease, for example*,* rheumatoid factor (RF), anti-citrullinated protein antibodies (ACPA) and the recently described antibodies directed against carbamylated proteins (anti-CarP antibodies) [1].

The citrullinated proteins recognized by ACPA arise due to the deimination of an arginine residue into citrulline by an enzyme, peptidyl arginine deaminase (PAD) [2]. We and others have shown that the presence of antibodies against citrullinated proteins/peptides (ACPA) measured as anti-CCP antibodies of immunoglobulin (Ig)G, IgA, and IgM isotypes, as well as RF, precedes the development of RA by a number of years [3-6]. More recently, we have also shown that an increasing number of ACPA specificities can be detected the closer the samples were collected before the onset of RA [7]. The ACPA specificities were initially restricted and without any obvious epitope profile, but over time expanded with epitope spreading and involved more specific responses, especially with regard to antibody reactivities against α-enolase (CEP-1/Eno5-21), fibrinogen (Fib)β36-52, and filaggrin (CCP-1/Fil307-324), when approaching the onset of symptoms [7,8]. The presence of ACPA in RA-patients has also been shown to predict a more severe disease [9-11].

In addition to deimination, another post-translational modification of proteins is carbamylation where preferentially lysines are converted to homocitrulline by a non-enzymatic process [12]. Anti-CarP antibodies have been identified in patients with RA [13]. Furthermore, these antibodies have been shown to be associated with the development of RA in patients with arthralgia [14] and were associated with a more severe disease course in ACPA-negative patients [13]. Similar to ACPA, anti-CarP antibodies have also been observed in individuals before the onset of clinical symptoms of RA [15].

In this study blood samples donated to the Medical Biobank of Northern Sweden by individuals prior to the onset of RA and of controls derived from the same population were analysed for anti-CarP antibodies. Although the presence of anti-CarP antibodies in samples from healthy subjects prior to clinical diagnosis of RA has already been shown in a relatively small Dutch cohort [15], we were now able to analyse samples from a larger cohort of individuals for the presence of anti-CarP antibodies in relation to antibodies against anti-CCP2 and several ACPA specificities. Furthermore, samples were collected before and after the onset of disease allowing a comparison between anti-CarP antibodies and ACPA fine-specificities with regard to their possible predictive values for development of RA as well as after subsequent disease progression.

## Methods

### Subjects

A case–control study was conducted based on individuals included in population surveys within the Medical Biobank of Northern Sweden and the Maternity cohort. The criteria for recruitment and collection and storage of blood samples have been described previously [6]. Cohorts included in the Medical Biobank are population based, and all adult individuals residing in the county of Västerbotten are continuously invited to participate. The Maternity cohort is a collection of serum samples obtained from pregnant women in northern Sweden who had undergone screening for immunity to rubella (6). The registers from the Medical Biobank and the Maternity cohort were co-analysed with the registers of patients with RA fulfilling the 1987 American Rheumatism Association classification criteria for RA [16] and attending the Department of Rheumatology, University Hospital in Umeå, with a known date for the onset of symptoms to identify individuals who had donated blood samples prior to the onset of symptoms of RA. In this study only samples donated less than 13 years before the onset of symptoms were included. The number of individuals identified and the procedure for excluding any sample has previously been described in detail [7]. Consequently, 252 individuals (58 men and 194 women) were included in this study, who were referred to as ‘pre-symptomatic individuals’ with a total of 423 (375 from the Medical Biobank and 48 from the Maternity cohort) blood samples at different time points. Forty-six individuals had contributed with two samples, 24 three samples, 13 four, seven with five and two with six samples. The median (interquartile range (IQR)) time pre-dating onset of symptoms using all samples was 5.2 (6.3) years and for the samples obtained closest to before disease onset it was 3.5 (4.9) years. Controls (N = 197) were randomly identified from the same cohorts within the register at the Medical Biobank and matched for age, sex and date of sampling. Of the pre-symptomatic individuals 192 were also sampled when they presented at the early arthritis clinic (Department of Rheumatology, University Hospital, Umeå) and were diagnosed with RA. The median (IQR) duration of symptoms before these patients were diagnosed following the onset of symptoms was 7 (5.8) months. Radiographs of the hands and feet of patients with RA (n = 181) were available at baseline and after 24 months; these radiographs were graded according to the Larsen score [9] and read in chronological order by two readers, who were blind to the antibody data. Radiological progression was defined as the increase of the Larsen score between baseline and 24 months, with the smallest detectable change less than four (calculated according to ref. [17]). All individuals were classified either as being a ‘non-smoker’ or an ‘ever-smoker’ (past or current). Demographic data of the cases and controls is presented in Table 1.

**Table 1:** **Demographic data for 252 individuals with 423 samples at a median (IQR) of 5.2 (6.3) years before the onset of symptoms of RA, patients with RA, and controls**

	**Controls (number = 197)**	**Pre-symptomatic individuals (number = 252)**	**RA-patients (number = 192)**
**Female sex, %**	84.3	77	75
**Median age, years (IQR)**	50.1 (20.2)	52 (17.4)^a^	57.5 (14.3)
**Ever smoker n/total (%)**	83/168 (49.4)	159/246 (64.6)	127/188 (67.6)

^a^Median age, as calculated for all samples when collected (number = 423). IQR, interquartile range; RA, rheumatoid arthritis.

The Regional Ethics Committee at the University Hospital, Umeå, Sweden, approved this study and all participants gave their written informed consent when donating blood samples.

### Analyses of anti-CarP antibodies and anti-CCP2 antibodies

The anti-CarP antibodies were determined by ELISA as described previously [13]. Briefly, Nunc Maxisorp plates (Nunc, Roskilde, Denmark) were coated overnight using carbamylated fetal calf serum (Ca-FCS) or non-modified FCS. After washing and blocking, the samples were added and allowed to bind overnight. Binding was determined using horseradish peroxidase (HRP)-conjugated rabbit-anti-human IgG (DAKO, Glostrup, Denmark) in combination with ABTS (2,2′-azino-bis(3-ethylbenzothiazoline-6-sulphonic acid). The investigators who carried out the anti-CarP ELISA tested the samples in a blind fashion. The cut-off level for anti-CarP antibodies was defined as the antibody reactivity expressed as the sum of optimal sensitivity and specificity, using receiver operating characteristic (ROC) curves, based on the concentrations in samples from the patients with RA included in this study and the controls from the Medical Biobank. The cut-off for positivity was set at 256.07 arbitrary units/mL (AU/mL) giving a specificity of 97%.

Detection of anti-CCP antibodies was successfully performed using ELISA according to the manufacturer’s instructions (Euro-Diagnostica, Malmö, Sweden). The cut off for positivity was set at 25 AU/mL.

### Multiplex assay

Serum and plasma samples were analysed for levels of ACPA specificities of the IgG isotype using a custom-made microarray based on the ImmunoCAP ISAC system (ThermoFischer/Phadia, Uppsala, Sweden) of the first version included on the platform [18]. Briefly, ten peptides representing the four candidate autoantigens citrullinated fibrinogen, a-enolase, vimentin and collagen type II: fibrinogen (Fib) α563-583 (citrullinated at position 573), Fibα580-600 (citrullinated at position 591), Fibβ62-81a (citrullinated at position 72), Fibβ62-81b (citrullinated at position 74), Fibβ36-52 (citrullinated at position 44), α-enolase (CEP-1/Eno5-21) ( citrullinated at position 10, 16), triple helical collagen type II (citC1 CII 359–369 with citrulline at 360 and 365), filaggrin (CCP-1/Fil307-324), vimentin (Vim) 2–17 (citrullinated at position 4, 12,13), and Vim60-75 (citrullinated at position 64, 69, 71), were analysed as previously described [7,18] (Additional file 1: Table S1). For all reactivities both the citrullinated and the native arginine-containing peptides were analysed and for all except one, citC1, the determined delta-value was used. The exception was due to the fact that the arginine containing citC1 is an autoantigen in itself [19]. The cut-off values were based on ROC curves analysed on the RA-patients and controls.

### Statistical analysis

For comparing continuous data between two groups the Mann–Whitney U test was used and the Kruskal Wallis test for several groups. Correlation analyses were performed using Spearman’s rank correlation test (r_s_). Logistic regression analyses were performed to identify associations between antibodies and disease development, presented as odds ratios (ORs) and 95% confidence intervals (95% CIs). Univariate analyses of variance were used to identify associations between factors and continuous data. Relationships between categorical data (positive versus negative) were compared using chi-square analysis or Fisher’s exact test as appropriate. Considering the study to be explorative, *P* values less than or equal to 0.05 were considered significant.

Statistical calculations were performed using SPSS for Windows, version 22. Sensitivity, specificity, odds ratios (ORs) and 95% confidence intervals (CIs) were calculated with the XLSTAT program (version 2014.1.04) in Microsoft Excel 2013 (Addinsoft).

## Results

### Levels of anti-CarP antibodies in pre-symptomatic individuals, patients and controls

**Figure 1:**
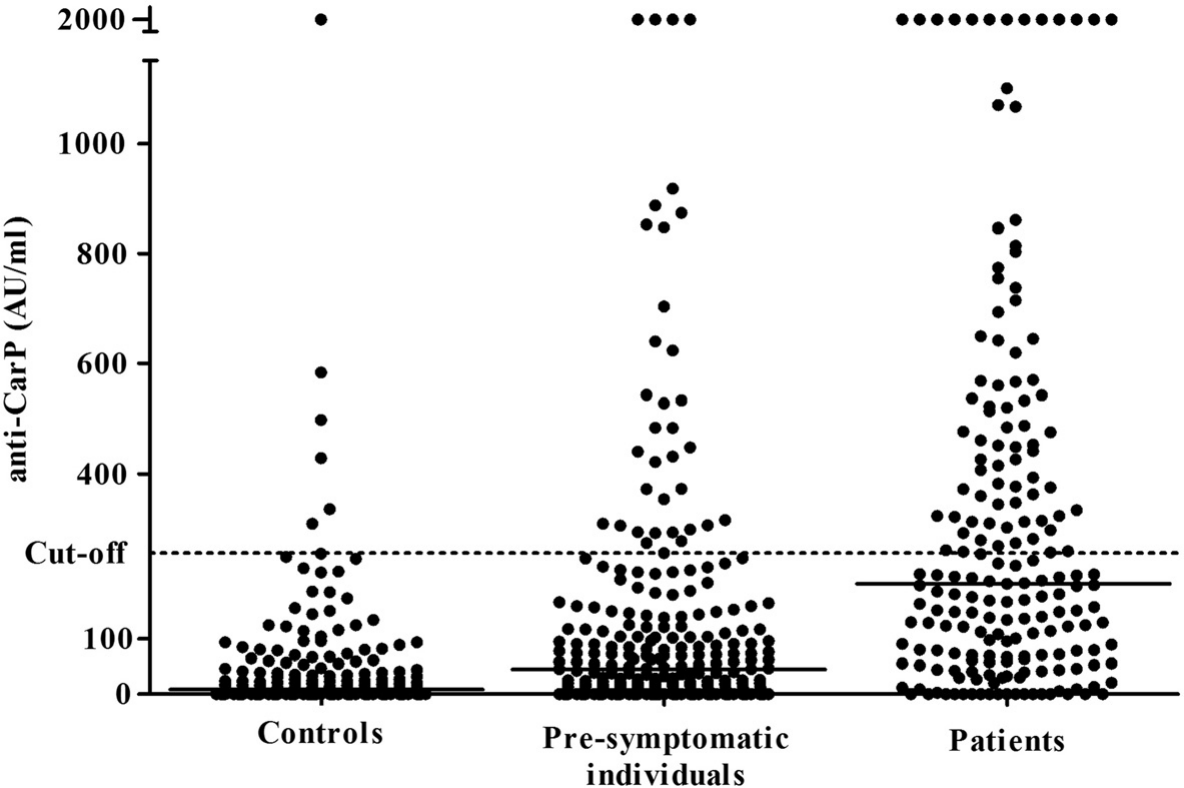
**Concentrations of anti-CarP antibodies in pre-symptomatic individuals closest to symptom onset (median (IQR) 3.5 (4.9) years) and in controls and patients with RA.** Anti-CarP, antibodies against carbamylated protein; IQR, interquartile range; RA, rheumatoid arthritis.

The levels of anti-CarP antibodies in the pre-symptomatic individuals were significantly increased compared with that of controls, namely, a median (IQR) of 44 (142) AU/mL and 8 (43) AU/mL, respectively (*P* <0.0001). Levels for the sample in each individual closest to onset of symptoms are presented in Figure 1 (Figure 1). The concentration after the onset of disease (median (IQR) 198 (379) AU/mL) was significantly increased compared with that for both the control subjects and pre-symptomatic individuals (*P* <0.0001, Kruskal-Wallis) (Figure 1).

### Frequency of anti-CarP antibody positivity in pre-symptomatic individuals, patients and controls

**Figure 2:**
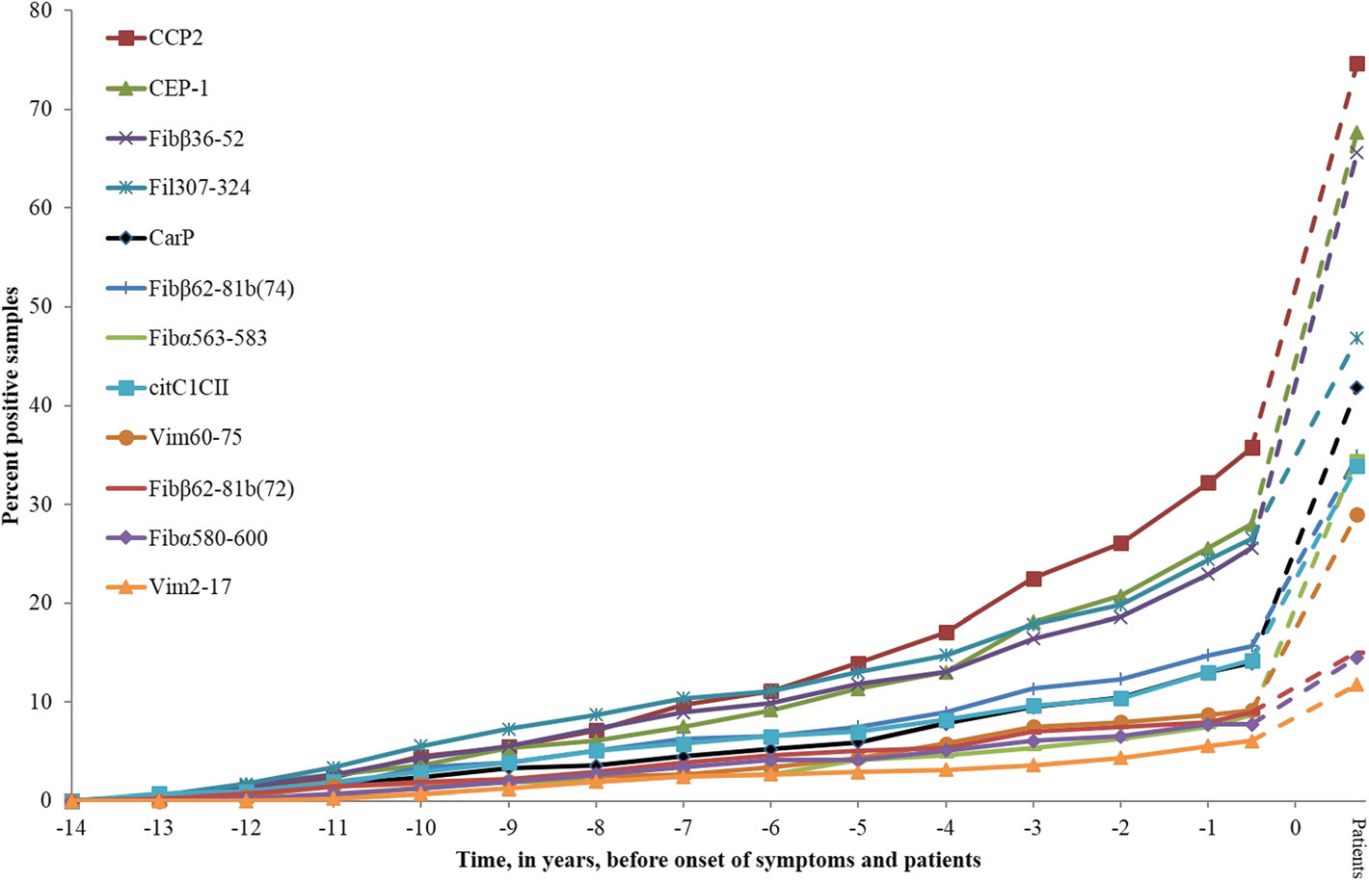
**Accumulated percentage of positivity of anti-CarP antibodies, the different ACPA specificities and anti-CCP2 antibodies.** ACPA, anti-citrullinated protein antibodies; anti-CarP, antibodies against carbamylated proteins; anti-CCP2, anti-cyclic citrullinated peptide 2.

The increased levels of anti-CarP antibodies in the pre-symptomatic individuals are also reflected in an increased frequency of anti-CarP positive individuals. The frequency of samples with anti-CarP antibodies was 13.9% (95% CI 11.0% to 17.6%) when all samples were included in the analysis, and the frequency of being ever positive for the antibody before onset of symptoms was 18.3% (95% CI 14.0% to 23.5%) and in the RA patients was 42.2% (95% CI 35.4% to 49.3%). The frequency of samples with positive anti-CarP antibody detection was higher the closer in time to onset of symptoms, for example, 21.8% (95% CI 14% to 32.3%) of the pre-symptomatic individuals had anti-CarP antibodies above the cut-off value less than two years before the onset of symptoms and 18.6% (95% CI 12.8% to 26.3) when analysed at less than three years before disease onset, although the gradual increase was not significant during the pre-dating period (Figure 2). Ever being positive for anti-CarP antibodies was associated with the development of RA in the pre-symptomatic individuals (OR = 7.1 95% CI 3.0 to 17.0). Adjustments for age, sex or ever being a smoker, did not affect the association between anti-CarP antibodies and disease development (data not shown). Furthermore, the association remained after adjusting for each ACPA fine specificities separately but not after adjusting for anti-CCP2 antibodies (OR = 2.4, 95% CI 0.9 to 6.7) (Table 2). However, after stratification for anti-CCP2 antibodies, anti-CarP antibodies were associated with disease development in anti-CCP2 negative pre-symptomatic individuals (OR = 3.4 95% CI 1.1 to 9.8) (data not shown). Analysis in patients with RA showed an association between anti-CarP antibodies and the RA disease also after adjusting for all ACPA specificities including anti-CCP2 antibodies (Table 2).

**Table 2:** **The association between anti-CarP antibodies and anti-CarP antibodies adjusted for ACPA and anti-CCP2, respectively**

	**Pre-symptomatic individuals** ^**a**^		**RA patients** ^**b**^	
**Antibodies against**	**OR**	**95% CI**	**OR**	**95% CI**
CarP	7.1	3.0-17.0	23.2	9.8-55.0
after adjustments for each antibody:				
CarP	2.4	0.9-6.9	3.8	1.2-12.2
CCP2	43.8	13.54-141.9	125.0	37.4-417.7
CarP	3.4	1.35-8.8	8.1	3.02-21.4
CEP-1	10.0	4.67-21.6	31.4	14.3-69.2
CarP	4.9	2.0-12.1	16.2	6.7-39.0
citC1	5.1	2.23-11.8	19.2	7.9-46.8
CarP	4.5	1.81-11.3	9.9	3.9-25.3
Fibß36-52	6.6	3.50-12.3	17.3	8.9-33.6
CarP	6.3	2.60-15.3	20.9	8.8-49.8
Fibß62-81a(72)	6.1	2.32-16.1	4.9	1.7-14.0
CarP	4.6	1.86-11.5	17.3	7.1-42.3
Fibß62-81b(74)	37.6	5.11-277.2	79.1	10.7-586.7
CarP	5.6	2.33-13.7	14.4	6.0-35.0
Fibα563-583	3.1	1.28-7.3	7.4	3.1-17.5
CarP	6.1	2.54-14.8	21.3	8.9-50.5
Fibα580-600	1.9	0.89-4.2	2.1	0.87-4.9
CarP	3.8	1.51-9.7	15.0	6.1-36.9
Fil307-324	12.8	5.40-30.3	19.2	7.9-46.9
CarP	5.9	2.42-14.2	17.3	7.2-41.4
Vim60-75	2.5	1.22-5.2	4.0	1.9-8.5
CarP	6.2	2.58-15.1	22.0	9.26-52.3
Vim2-17	3.1	1.24-8.0	3.6	1.333-9.9
CarP	3	1.2-8.1	12.2	4.9-30.4
CCP2+ and all ACPA	7.2	4.7-11.2	9.7	5.7-16.5

^a^Calculated as ever being positive, n = 253; ^b^number = 192. ACPA, anti-citrullinated protein antibodies; anti-CarP, antibodies against carbamylated proteins; anti-CCP2, anti-cyclic citrullinated peptide 2; CI, confidence interval; fib, fibrinogen; OR, odds ratio; RA, rheumatoid arthritis; vim, vimentin.

### Relationships between anti-CarP antibodies and ACPA specificities and anti-CCP2 antibodies

**Figure 3:**
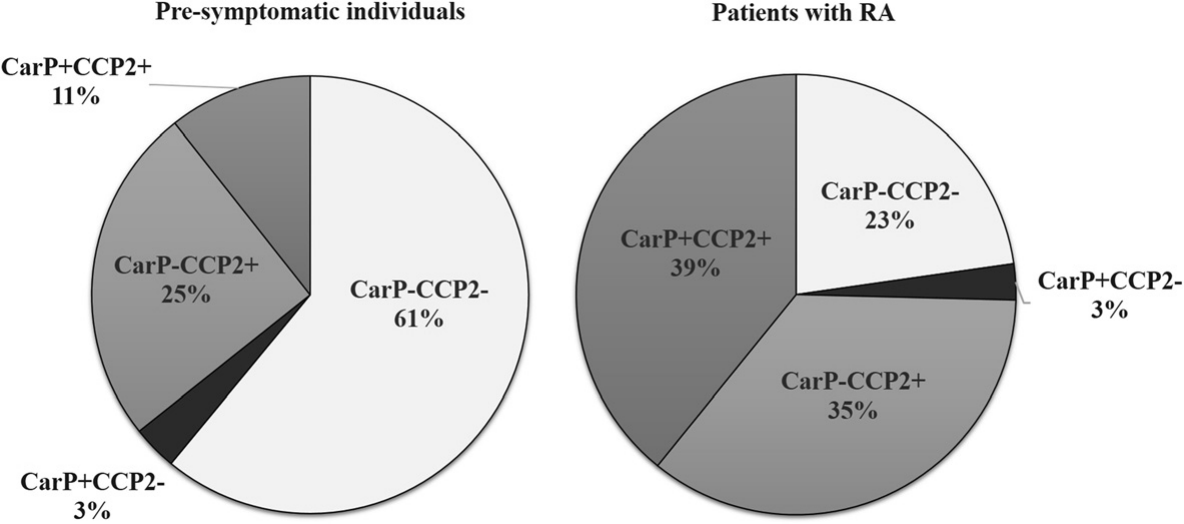
**The relative distribution in percentage of the combinations of positivity and negativity of anti-CarP and anti-CCP2 antibodies illustrated by pie charts.** anti-CarP, antibodies against carbamylated proteins; anti-CCP2, anti-cyclic citrullinated peptide 2.

The presence of anti-CarP antibodies, anti-CCP2 and several ACPA specificities was investigated in order to describe populations of patients and pre-symptomatic individuals with overlapping and unique antibody specificities (Table 3). Among the pre-symptomatic individuals negative for any of the individual ACPA specificities 8.1% to 13.5% were positive for anti-CarP antibodies, the lowest frequency being for anti-CEP-1 and the highest for anti-Fibß62-81a (72). When selecting samples negative for all ACPA specificities irrespective of anti-CCP2 antibodies, the frequencies of positivity for anti-CarP antibodies were 2.8%, 5.8% and 13.3% in controls, pre-symptomatic individuals and RA-patients, respectively (for further details on the relationships to sub specificities, see Table 3). The distribution of the combinations of anti-CarP and anti-CCP2 before and after disease onset showed a relative increase of double positivity after onset compared with the distribution before onset where the number of double negativity dominated (Figure 3). The relative distribution of the combinations of one antibody negative and the other antibody positive (anti-CarP+/anti-CCP- and CarP-/anti-CCP+) were fairly unchanged (Figure 3).

**Table 3:** **Frequency of positivity for anti-CarP antibodies in relation to positive/negative ACPA or anti-CCP2 antibodies**

	**Pre-symptomatic individuals**		**RA patients**	
**Antibodies against**	**Number (%)**	**Anti- CarP + number (%)**	**Number (%)**	**Anti- CarP + number (%)**
All individuals	423 (100)	59 (13.9)	188 (100)	81 (42.2)
CCP2 +	151 (35.7)	45 (29.8)	141 (74.6)	74 (52.5)
CCP2 -	272 (64.3)	14 (5.1)	48 (25.4)	5 (10.4)
CEP-1 +	116 (28)	32 (27.6)	128 (68.1)	68 (53.1)
CEP-1 -	299 (72)	24 (8.1)	60 (31.9)	11 (18.3)
CitC1 +	59 (14.2)	18 (30.5)	63 (33.5)	38 (60.3)
CitC1 -	356 (85.8)	38 (10.7)	125 (66.5)	41 (32.8)
Fibß36–52 +	101 (24.4)	24 (23.8)	122 (64.9)	66 (54.1)
Fibß36–52 -	313 (75.6)	31 (9.9)	66 (35.1)	13 (19.7)
Fibß62-81a(72)+	38 (9.2)	5 (13.2)	28 (14.9)	16 (57.1)
Fibß62-81a(72) -	377 (90.8)	51 (13.5)	160 (85.1)	63 (39.4)
Fibß62-81b(74)+	65 (15.7)	20 (30.8)	67 (35.6)	36 (53.7)
Fibß62-81b(74) -	350 (84.3)	36 (10.3)	121 (64.4)	43 (35.5)
Fibα580-600 +	32 (7.7)	9 (28.1)	27 (14.4)	16 (59.3)
Fibα580-600-	383 (92.3)	47 (12.3)	161 (85.6)	63 (39.1)
Fibα563-583 +	36 (8.7)	9 (25)	65 (34.6)	43 (66.2)
Fibα563-583 -	379 (91.3)	47 (12.4)	123 (65.4)	36 (29.3)
Fil307-324 +	110 (26.5)	27 (24.5)	88 (46.8)	48 (54.5)
Fil307-324 -	305 (73.5)	29 (9.5)	100 (53.2)	31 (31)
Vim 2–17 +	25 (6)	4 (16)	22 (11.7)	10 (45.5)
Vim 2–17 -	390 (94)	52 (13.3)	166 (88.3)	69 (41.6)
Vim 60–75 +	38 (9.2)	9 (23.7)	56 (29.8)	35 (62.5)
Vim 60-75-	377 (90.8)	47 (12.5)	132 (70.2)	44 (33.3)

The concentration of anti-CarP antibodies correlated with those of anti-CCP2 antibodies both in the pre-symptomatic individuals and in RA patients (r_s_= 0.31 and 0.52, respectively, *P* <0.001 for both). Additionally there were correlations between the concentrations of anti-CarP antibodies and the different ACPA specificities in both pre-symptomatic individuals (r_s_ = 0.18 to 0.34, *P* <0.0001 to 0.04) and RA patients (r_s_ = 0.15 to 0.46, *P* <0.0001 to 0.04) with the exception of Vim2-17 and Fibα580-600(591) in both groups and Fibß62-81a(72) in the pre-symptomatic individuals.

There were no significant differences between anti-CarP antibodies or anti-CCP2 antibodies or ACPA specificities in the predating time when the antibodies were first detected positive for any of the antibodies (data not shown). In 20 and 42 individuals anti-CCP2 antibodies and any ACPA specificity, respectively, preceded positivity for anti-CarP antibodies. In five and two individuals anti-CarP antibody positivity preceded the presence of anti-CCP2 antibodies and any ACPA specificity, respectively.

### The presence of anti-CarP antibodies is associated with radiological damage, especially in anti-CCP2 negative individuals

Positivity for anti-CarP antibodies in the pre-symptomatic individuals was associated with increased radiological damage as detected by a higher Larsen score at baseline, that is, when RA was diagnosed, the median (IQR) value for the Larsen score was 9.0 (6.5) compared with 5.0 (9.0) (*P* = 0.003) in anti-CarP antibody negative individuals. This association remained after adjustment for anti-CCP2 antibodies. Anti-CarP antibodies analysed after disease onset were also associated with radiological progression and outcome calculated as Larsen score at baseline in the patients with early RA (ß = 2.15, 95% CI 0.40 to 3.90, *P* = 0.017) at 24 months (ß = 4.49, 95% CI 1.67 to 7.32, *P* = 0.002) and also with the radiological progression (ß = 2.44, 95% CI 0.53 to 4.35, *P* = 0.013). The outcome at 24 months remained significant after adjustment for the Larsen score at baseline (ß = 1.89, 95% CI 0.02 to 3.75, *P* = 0.048). Stratification for anti-CCP2 antibodies revealed that the association of anti-CarP antibodies was strengthened in anti-CCP2 negative patients at baseline (ß = 5.38, 95% CI 0.94 to 39.82, *P* = 0.019), after 24 months (ß = 8.57, 95% CI 3.36 to 13.79, *P* = 0.002) and the radiological progression (ß = 2.3.02, 95% CI 0.17 to 5.87, *P* = 0.038), and non-significant in anti-CCP2 positive patients. The pattern was similar when stratified for the presence or absence of the triplet of the most frequently occurring ACPA fine specificities (that is*,* anti-CEP-1, −Fibß36-52 and – Fil307-324) in anti-CarP positive patients (24 months; (ß = 4.25, 95% CI 0.70 to 7.80, *P* = 0.019). However, when stratified for these three ACPA specificities separately there were differences between them. The presence of anti-CarP antibodies was associated with a higher Larsen score in anti-Fibß36-52 and anti-CEP-1 antibody positive patients at baseline, and after 24 months, whilst more radiological damage was found in anti-CarP positive and anti-Fil307-324 antibody negative patients (data not shown). The presence of anti-CCP2 antibodies as well as antibodies against CEP-1, Vim60-75 and CCP1/Fil307-324 was also significantly associated with radiological findings at 24 months and with radiological progression versus being negative, although to a lower extent compared with analysis for anti-CarP antibodies (data not shown). None of the relationships of anti-CCP2, antibodies against CEP-1, Vim60-75 and Fil307-324 remained significant in separate univariate analyses of variance including anti-CarP antibodies that were significantly associated in all analyses (data not shown). Adding the information on positivity for anti-CCP2 antibodies to the presence of anti-CarP antibodies further increased the association to radiological findings at 24 months (ß = 5.4, 95% CI 2.01 to 8.78) and for radiological progression (ß = 3.05. 95% CI 0.75 to 5.35).

## Discussion

In this relatively large collection of samples from individuals before they had any symptoms of a subsequent joint disease, increased levels of anti-CarP antibodies were found. The levels were also further increased when these individuals had developed RA. The levels of anti-CarP antibodies correlated with the levels of most of the ACPA specificities and with that of anti-CCP2, both before and after disease development. These findings are consistent with previous findings but now also include several ACPA specificities [15].

The time point for the first antibody to be detectable in samples from the pre-symptomatic individuals was comparable for the anti-CarP antibodies and the different ACPA specificities and to anti-CCP2 antibodies. There was a gradual increase in the frequency of positivity for the anti-CarP antibodies during the time pre-dating that peaked at the onset of symptoms. The frequency of these antibodies was lower than the three most frequently occurring ACPA fine specificities (anti-CEP-1, −Fibß36-52 and –Fil307-324 peptides), and also than anti-CCP2 antibodies during the pre-dating period. However, analyses of samples closer to symptom onset showed that the frequency of anti-CarP antibodies almost reached the level of Fil307-324 peptide antibodies.

In our previous study we found a clear and significant increase during the predating time of antibodies against CEP-1, Fibß36-52 and Fil307-324 peptide and also of anti-CCP2 antibodies [7]. Antibodies against Vim60-75 increased shortly before the disease developed. The increasing frequency of anti-CarP antibodies initially appeared to be more similar to antibodies against Vim60-75 but shortly before the onset of symptoms more closely reached the level of antibodies against Fil307-324 peptide.

Even though in our study 3% of the RA patients are single positive for anti-CarP antibodies and 36% are single positive for anti-CCP there is also a substantial portion of patients (39%) who are double positive and, therefore, cross-reactivity towards citrullinated and carbamylated proteins must be considered. Previous experiments indicated that, next to cross-reactive antibodies, antibodies only reacting with either citrullinated or carbamylated proteins were also present in RA [20]. In addition results from a recent study by Jiang *et al*. [21] on two large cohorts of RA found similar distributions regarding anti-CarP and anti-CCP antibodies and also found completely different HLA associations for anti-CarP and anti-CCP antibodies. In addition, the association between anti-CCP antibodies and smoking was not found for anti-CarP antibodies. Collectively, these findings do not favour the idea that anti-CarP antibodies only represent cross-reactivity to citrullinated antigen. It is likely that both cross-reactive and non-cross-reactive antibodies co-exist.

Our results on the appearance of anti-CarP antibodies in samples collected years before the onset of symptoms confirm previous observations [14], although the number of samples available for this study is larger and not restricted to those individuals identified as being positive in their last sample before being diagnosed with RA. Our cohort is only restricted to samples donated less than 13 years before onset of symptoms. Anti-CarP antibodies were strongly associated with RA both among the pre-symptomatic individuals and among RA-patients (OR = 7.1 and 23.2, respectively). However, when adjusted for the presence of anti-CCP2, the association was non-significant (OR = 2.4 95% CI 0.9 to 6.7) in the pre-symptomatic individuals but still significant in RA patients (OR = 3.8 95% CI 1.2 to 12.2). However, after stratification for anti-CCP2 antibodies the association of anti-CarP antibodies and disease development remained significant in anti-CCP2 negative individuals irrespective of adjustments for each separate ACPA fine specificities or analysed as one group. Anti-CarP antibodies were associated with disease development when adjusted for all of the ACPA fine specificities, each analysed separately or in combination as one group with anti-CCP2 antibodies. Only 3% of the pre-symptomatic individuals and RA patients, respectively, were anti-CarP positive anti-CCP2 negative when the group of individuals before symptom onset or of RA patients were analysed. However, we found that if we only considered a group of anti-CCP2 negative patients, the frequency of anti-CarP positive antibodies was 10.4%. This information could be useful in a clinical situation to diagnose an arthritis as RA when the anti-CCP2 test is negative.

The present cohort provided the opportunity to evaluate the radiological findings when the symptoms had developed and the individuals were diagnosed with RA and at a follow-up after 24 months. We found that the presence of anti-CarP antibodies before onset of symptoms of the disease predicted the radiological findings at baseline. Furthermore, we were able to confirm the findings by Shi and colleagues [13] that the presence of anti-CarP antibodies is associated with the rate of radiological destruction. In the present study this was already apparent at baseline, after 24 months and also calculated as the change over time. The association of anti-CarP antibody positivity was particularly evident in anti-CCP2 negative or Fil307-324 peptide negative individuals. The associations of anti-CCP2, and some of the ACPA specificities (CEP-1, Vim60-75 and Fil307-324) were also associated with radiological findings at 24 months and with the change over time but they did not remain significant after adjusting for presence of anti-CarP antibodies.

## Conclusions

We conclude that the anti-CarP antibodies are present years before the onset of symptoms of RA. These antibodies are also associated with disease development after adjusting for the presence of different ACPA fine specificities and in anti-CCP2 negative individuals. We are also able to confirm that the presence of these antibodies is related to radiological destruction at diagnosis and to the radiological progression observed once the disease has developed.

## Additional file

Additional file 1: Table S1. Amino acid sequences of the analysed citrullinated antigens.

## Abbreviations

ACPA: anti-citrullinated protein antibodies
anti-CarP: antibodies against carbamylated proteins
anti-CCP: anti-cyclic citrullinated peptide antibodies
CI: confidence interval
ELISA: enzyme-linked immunosorbent assay
Fib: fibrinogen
HLA-SE: human leukocyte antigen – shared epitope
IQR: interquartile range
OR: odds ratio
RA: rheumatoid arthritis
ROC: receiver operating characteristic
vim: Vimentin
