# Supplementary material for: Anti-carbamylated protein antibodies in the pre-symptomatic phase of rheumatoid arthritis, their relationship with multiple anti-citrulline peptide antibodies and association with radiological damage
Source: Arthritis Res Ther. 2015 Feb 7;17(1):25. doi: 10.1186/s13075-015-0536-2 (PMC4350267; doi:10.1186/s13075-015-0536-2)
Supplement: Additional file 1: Table S1. — Amino acid sequences of the analysed citrullinated antigens. [file 13075_2015_536_MOESM1_ESM.docx]

| Additiona file 1: Table S1.  Amino acid sequences of the analysed citrullinated antigens | | | |
| --- | --- | --- | --- |
| **Citrullinated antigen** | Citrullinated position | **Amino acid sequence** | Ref. |
| Fibrinogen α563-583 | 573 | HHPGIAEFPS(cit)GKSSSYSKQF | 1 |
| Fibrinogen α580-600 | 591 | SKQFTSSTSYN(cit)GDSTFESKS | 1 |
| Fibrinogen ß62-81a | 72 | APPPISGGGY(cit)ARPAKAAAT | 1 |
| Fibrinogen ß62-81b | 74 | APPPISGGGYRA(cit)PAKAAAT | 1 |
| Fibrinogen ß36-52 | 44 | NEEGFFSA(cit)GHRPLDKK | 2 |
| Citrullinated alpha-enolase peptide (CEP-1/Eno5-21) | 10, 16 | CKIHA(cit)EIFDS(cit)GNPTVEC (cyclic) | 3, 4 |
| Citrullinated triple-helical collagen type II peptide C1 (359-369) (citC1^III^) | 360, 365 | (GPO)5-GA(cit)GLTG(cit)PGDA(GPO)2-  GKKYG | 5,6 |
| Filaggrin (CCP-1/Fil307-324 | 13 | SHQEST(cit)GRSRGRSGRSGS (cyclic) | 7, 8 |
| Vimentin 2-17 | 4, 12,13 | ST(cit)SVSSSSY(cit)(cit)MFGG | 9 |
| Vimentin 60-75 | 64, 69, 71 | VYAT(cit)SSAV(cit)L(cit)SSVP | 10, 2 |

1. Hermansson, M., et al MS analysis of rheumatoid arthritic synovial tissue identifies specific citrullination sites on fibrinogen. Proteomics Clin Appl 2010; **4**: 511-8.

2. Verport KN, et al., Fine specificity of the anti-citrullinated protein antibody response is influenced by the shared epitope alleles. Arthritis Rheum 2007; 56: 3949-3952.

3. Lundberg K, et al., Antibodies to citrullinated alpha-enolase peptide 1 are specific for rheumatoid arthritis and cross-react with bacterial enolase. Arthritis Rheum 2008;58:3009-3019.

4. Kinloch A, et al., Synovial fluid is a site of citrullination of autoantigens in inflammatory arthritis. Arthritis Rheum 2008;58:2287-95.

5. Burkhardt H, et al., Epitope-specific recognition of type II collagen by rheumatoid antibodies is shared with recognition by antibodies that are arthritogenic in collagen-induced arthritis in the mouse. Arthritis Rheum 2002;46:2339-2348.

6. Uysal H, et al., Structure and pathogenicity of antibodies specific for citrullinated collagen type II in experimental arthritis. J Exp Med 2009;206:449-62. Epub 2009 Feb 9.

7. Schellekens GA, et al., Citrulline is an essential constituent of antigenic determinants recognized by rheumatoid arthritis-specific autoantibodies. J Clin Invest 1998;101:273-281.

8. Schellekens GA, et al.,The diagnostic properties of rheumatoid arthritis antibodies recognizing a cyclic citrullinated peptide. Arthritis Rheum 2000;43:155-63.

9. Snir O, et al., Antibodies to several citrullinated antigens are enriched in the joints of rheumatoid arthritis patients. Arthritis Rheum 2010;62:44-52.

10. Vossenar E et al.,. Rheumatoid arthritis specific anti-Sa antibodies target citrullinated vimentin. Arthritis Res Ther 2004;6:R142-50. Epub 2004 Feb 5.
